# Yeokwisan Attenuates Reflux-Induced Esophageal Injury and Suppresses Macrophage Inflammatory Responses Associated with MAPK/PI3K-Akt and NF-κB/COX-2 Signaling

**DOI:** 10.3390/ph19091422

**Published:** 2026-09-09

**Authors:** Kyuhyung Jo, Aejin Kim, So Yeon Kim, Dong-Seon Kim, Chan-Sik Kim, Eunjung Son, Yun Mi Lee

**Affiliations:** 1 Korean Medicine Convergence Research Division, Korea Institute of Oriental Medicine, 1672 Yuseong-daero, Yuseong-gu, Daejeon 34054, Republic of Korea; jopd7414@kiom.re.kr (K.J.); kim3216390@kiom.re.kr (A.K.); chskim@kiom.re.kr (C.-S.K.); 2 Korean Convergence Medical Science Major, Campus of Korea Institute of Oriental Medicine, University of Science & Technology, 1672 Yuseong-daero, Yuseong-gu, Daejeon 34054, Republic of Korea; dskim@kiom.re.kr; 3 Korean Medicine Science Research Division, Korea Institute of Oriental Medicine, 1672 Yuseong-daero, Yuseong-gu, Daejeon 34054, Republic of Korea; kimsy0913@kiom.re.kr (S.Y.K.); ejson@kiom.re.kr (E.S.)

**Keywords:** reflux esophagitis, Yeokwisan, oxidative stress, inflammatory responses, MAPK, PI3K/Akt, NF-κB

## Abstract

**Background/Objectives**: Reflux esophagitis (RE) is a chronic inflammatory disorder caused by reflux of gastric contents into the esophagus, leading to mucosal injury and epithelial barrier dysfunction. Although proton pump inhibitors are widely used, persistent inflammation and oxidative stress remain important therapeutic challenges. Yeokwisan (YWS) is prescribed for gastroesophageal reflux disease and functional dyspepsia; however, its protective mechanisms against RE remain unclear. This study investigated the protective and anti-inflammatory effects of YWS. **Methods**: We integrated network pharmacology analysis, mechanistic studies in lipopolysaccharide (LPS)-stimulated RAW264.7 macrophages, and efficacy evaluation in a rat model of acute RE induced by pyloric and forestomach ligation. **Results**: Network pharmacology identified 174 common targets between YWS and RE and 23 hub genes through protein–protein interaction network and topological analyses, implicating PI3K/Akt, MAPK, Ras, HIF-1, chemokine, and other inflammation-related signaling pathways. In LPS-stimulated RAW264.7 macrophages, YWS significantly reduced intracellular reactive oxygen species, nitric oxide, TNF-α, and IL-6 production without inducing cytotoxicity and suppressed MAPK, PI3K/Akt, and NF-κB activation. In vivo, YWS attenuated acute reflux-induced esophageal mucosal injury and histopathological alterations, restored esophageal superoxide dismutase activity, reduced malondialdehyde levels, and lowered serum TNF-α, IL-6, and IL-1β levels. YWS also suppressed NF-κB/IκB activation and COX-2 expression in esophageal tissue. **Conclusions**: YWS exerted protective effects against acute reflux-induced esophageal injury and anti-inflammatory effects in activated macrophages, accompanied by attenuation of oxidative stress and inflammation-associated signaling. These findings support further investigation of YWS for reflux-associated esophageal inflammation.

## 1. Introduction

Reflux esophagitis (RE), a chronic gastrointestinal disorder characterized by the reflux of gastric acid and other gastric contents into the esophagus, results in irritation and inflammation of the esophageal mucosa. Patients with RE commonly experience symptoms such as heartburn, regurgitation, dysphagia, and chest pain, which can significantly impair the patients’ quality of life. Persistent mucosal inflammation and injury may result in complications, including esophageal stricture, erosive esophagitis, and Barrett’s esophagus, a premalignant condition associated with an increased risk of esophageal adenocarcinoma [1,2,3]. The pharmacological management of RE primarily focuses on suppressing gastric acid secretion. Proton pump inhibitors (PPIs), which inhibit the gastric H^+^/K^+^-ATPase proton pump in parietal cells, are widely used as first-line therapy owing to their potent acid-suppressive effects and ability to promote mucosal healing. In addition to PPIs, histamine H2 receptor antagonists (H2RAs), antacids, and mucosal protective agents are adjunct therapies.

Despite their clinical effectiveness, approximately 30–40% of patients with RE exhibit incomplete responses to PPI therapy or experience symptom relapse after treatment discontinuation. Moreover, long-term PPI use has raised concerns regarding potential adverse effects. Importantly, gastric acid suppression alone may not fully prevent mucosal injury because non-acidic reflux components, including bile acids, pepsin, and inflammatory mediators, can contribute to esophageal damage [4,5,6]. Esophageal inflammation is not merely a secondary consequence of acid exposure but is central to the pathogenesis and progression of gastroesophageal reflux disease (GERD). Contact between refluxate and the esophageal epithelium triggers immune cell infiltration and promotes the production of inflammatory cytokines, such as interleukin-6 (IL-6), IL-8, and tumor necrosis factor-α (TNF-α). These inflammatory responses are closely associated with the activation of intracellular signaling pathways, including nuclear factor-κB (NF-κB), mitogen-activated protein kinase (MAPK), and phosphoinositide 3-kinase/protein kinase B (PI3K/Akt) pathways, which contribute to mucosal injury and disease progression [7,8,9]. Therefore, complementary therapeutic approaches targeting inflammatory responses, oxidative stress, and epithelial barrier dysfunction may be more effective than acid suppression alone in treating RE.

Yeokwisan (YWS), a multi-herbal formulation, is clinically prescribed for patients with GERD and refractory functional dyspepsia (FD). Previous studies have demonstrated its gastroprotective, antioxidant, and anti-inflammatory activities in experimental models of gastrointestinal disorders [10]. In our previous study, we quantified several chemical constituents of YWS and demonstrated its antioxidant activity [11]. YWS attenuated gastric mucosal injury and exerted anti-inflammatory effects in animal models of alcohol- and restraint-stress-induced gastritis [12]. However, the precise molecular mechanisms underlying its protective effects, particularly in RE, remain unclear. Therefore, the aim of the present study was to investigate the protective effects of YWS against RE and to characterize its anti-inflammatory activity by integrating network pharmacology analysis, mechanistic studies in lipopolysaccharide (LPS)-stimulated RAW264.7 macrophages, and efficacy evaluation in a rat model of RE induced by pyloric and forestomach ligation.

## 2. Results

### 2.1. Network Pharmacology-Based Analysis of YWS Action Against RE

**Figure 1:**
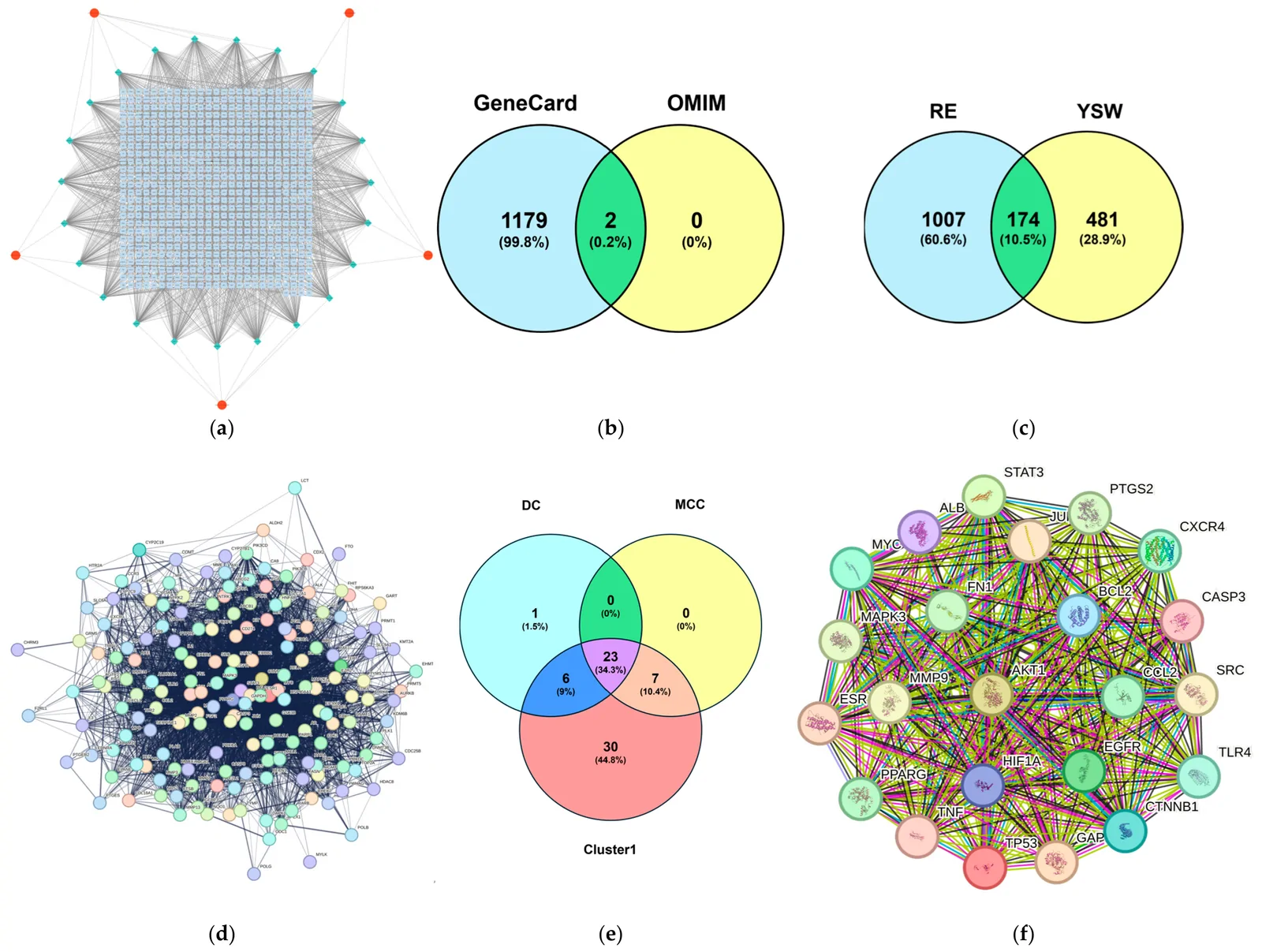

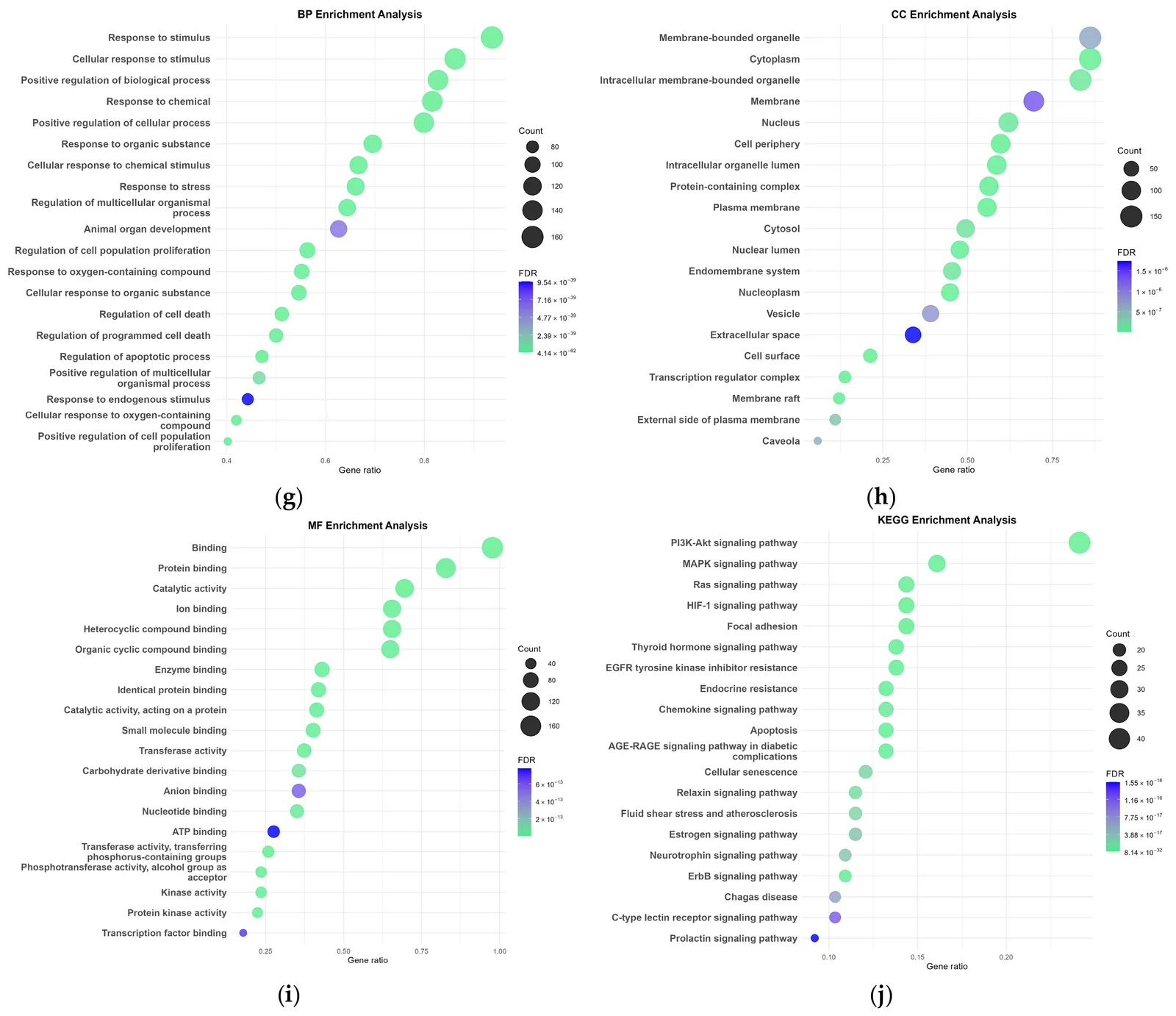
Network pharmacology analysis of YWS action against reflux esophagitis. (**a**) Compound–target network of YWS. Red circular nodes represent the herbal components of YWS, green diamond-shaped nodes represent the constituent compounds of the herbs, and light-blue rectangular nodes represent target genes. (**b**) RE-related target genes identified using GeneCards and OMIM. (**c**) A Venn diagram showing 174 common genes among RE and YWS targets. (**d**) A protein–protein interaction network of the common targets generated using STRING. (**e**) Venn diagram for the selection of core hub genes based on Degree Centrality (DC), Maximal Clique Centrality (MCC), and Cluster 1. (**f**) Network analysis for the 23 core genes. (**g**–**i**) GO enrichment analysis, including biological process (BP), cellular component (CC), and molecular function (MF) terms. (**j**) KEGG pathway enrichment analysis for the core targets.

A comprehensive network pharmacology analysis was performed to elucidate the pharmacological mechanisms underlying the protective effects of YWS against RE. Initially, 1195 potential targets for YWS were identified using SwissTargetPrediction, PharmMapper, and STITCH, and an herb-compound–target network was constructed to visualize these interactions (Figure 1a; enlarged view in Figure S1). Simultaneously, 1181 RE-related targets were retrieved from the OMIM and GeneCards databases (Figure 1b). By intersecting the YWS-predicted targets with RE-associated genes, 174 common targets were identified as candidate genes for further investigation (Figure 1c). Detailed information on the YWS components, predicted YWS targets, RE-related targets, and their 174 common targets is provided in Supplementary Data Tables S1–S4. Subsequently, a protein–protein interaction network was constructed using the STRING database to explore the interaction landscape of these 174 common targets, resulting in a network with 174 nodes and 4474 edges (Figure 1d; enlarged view in Figure S2). To identify core predicted targets, we performed topological analysis using Cytoscape (v.3.10.4). By identifying the intersection of top-ranked genes using Degree Centrality, MCC via the CytoHubba plugin, and MCODE Cluster 1, 23 hub genes were ultimately selected as core predicted targets associated with the effects of YWS (Figure 1e,f). The complete protein–protein interaction interaction data and hub gene screening results are provided in Tables S5–S7, respectively. Among these, *AKT1*, *MAPK3*, *STAT3*, *TNF*, *PTGS2*, *EGFR*, and *HIF1A* exhibited high connectivity within the network and were identified as highly connected predicted hub genes associated with inflammation- and stress-related signaling pathways relevant to RE pathogenesis.

Although numerous GO terms and KEGG pathways were enriched, only pathways containing at least three hub genes were prioritized for further interpretation. GO analysis yielded 900 BP terms, 65 CC terms, and 73 MF terms, whereas KEGG analysis identified 74 significantly enriched pathways. The most representative GO terms and KEGG pathways associated with RE pathophysiology are summarized in Figure 1g–j, whereas the complete GO and KEGG enrichment results are provided in Table S8. Twenty-three core genes were mainly associated with PI3K-Akt, MAPK, Ras, HIF-1, focal adhesion, chemokine signaling, and apoptosis pathways. The enrichment analysis suggested the involvement of inflammatory signaling, oxidative stress responses, epithelial apoptosis, and epithelial barrier dysfunction in the predicted YWS–RE interaction network. In particular, *AKT1* and *MAPK3* were among the highly connected predicted hub genes associated with the PI3K/Akt and MAPK signaling pathways, respectively. These findings suggest that the predicted effects of YWS may involve multiple inflammation-associated pathways, particularly PI3K/Akt-, MAPK-, and NF-κB-related signaling networks.

### 2.2. YWS Reduces Intracellular ROS Levels and Inflammatory Mediator Production in LPS-Stimulated RAW264.7 Macrophages

**Figure 2:**
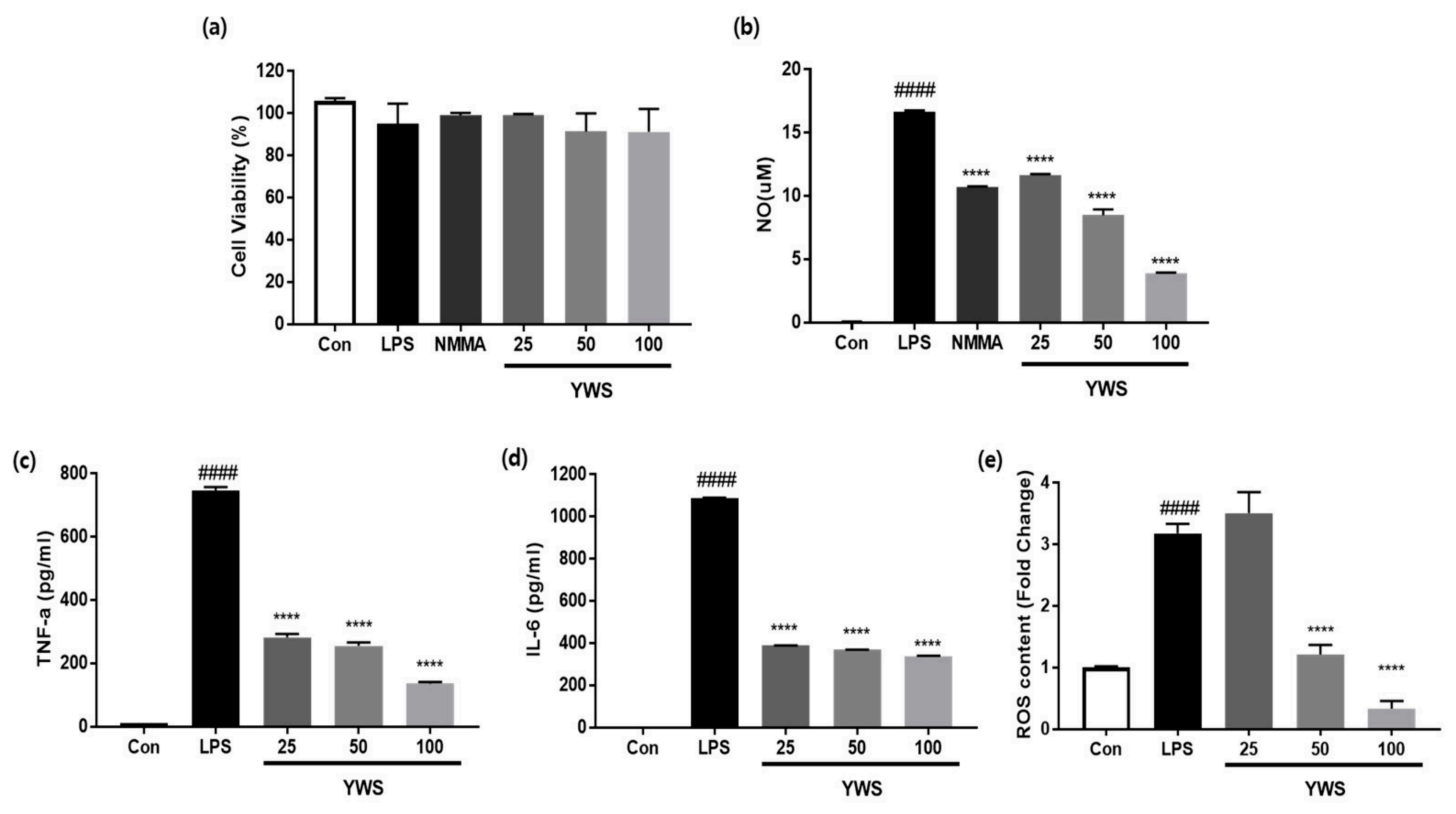
Effects of YWS on nitric oxide and pro-inflammatory cytokine production in LPS-stimulated RAW264.7 macrophages. (**a**) Cell viability was evaluated using the Cell Counting Kit-8 assay, and (**b**) nitric oxide (NO) production, pro-inflammatory cytokine levels, including (**c**) TNF-α and (**d**) IL-6, and (**e**) intracellular reactive oxygen species (ROS) generation were measured following experimental treatment (*n* = 3). #### *p* < 0.0001 vs. untreated control group (Con); **** *p* < 0.0001 vs. LPS-treated group (LPS).

RAW264.7 macrophages were treated with YWS followed by LPS stimulation to evaluate the anti-inflammatory effects of YWS. YWS did not exhibit cytotoxic effects at the tested concentrations, as confirmed by cell viability analysis (Figure 2a). As shown in Figure 2b–d, LPS markedly increased NO production, whereas YWS significantly suppressed LPS-induced NO production (*p* < 0.0001). Similarly, the levels of pro-inflammatory cytokines, including TNF-α and IL-6, were significantly elevated following LPS stimulation; however, YWS dose-dependently reduced TNF-α and IL-6 levels (*p* < 0.0001). Collectively, these results demonstrate that YWS exerts anti-inflammatory effects in macrophages by inhibiting NO and pro-inflammatory cytokine production without inducing cytotoxicity.

To further investigate the effects of YWS on intracellular oxidative stress, ROS production was measured in LPS-stimulated RAW264.7 macrophages. As shown in Figure 2e, LPS stimulation significantly increased intracellular ROS levels compared with those in the control group. However, treatment with YWS markedly reduced ROS production in LPS-stimulated RAW264.7 cells. In particular, YWS treatment at 50 and 100 μg/mL significantly suppressed ROS levels, indicating a strong inhibitory effect on LPS-induced oxidative stress. These results suggest that YWS effectively attenuates intracellular ROS generation in macrophages under inflammatory conditions.

### 2.3. YWS Suppresses LPS-Induced Inflammatory Signaling by Inhibiting MAPK- and PI3K/Akt-Mediated NF-κB Activation in RAW264.7 Macrophages

**Figure 3:**
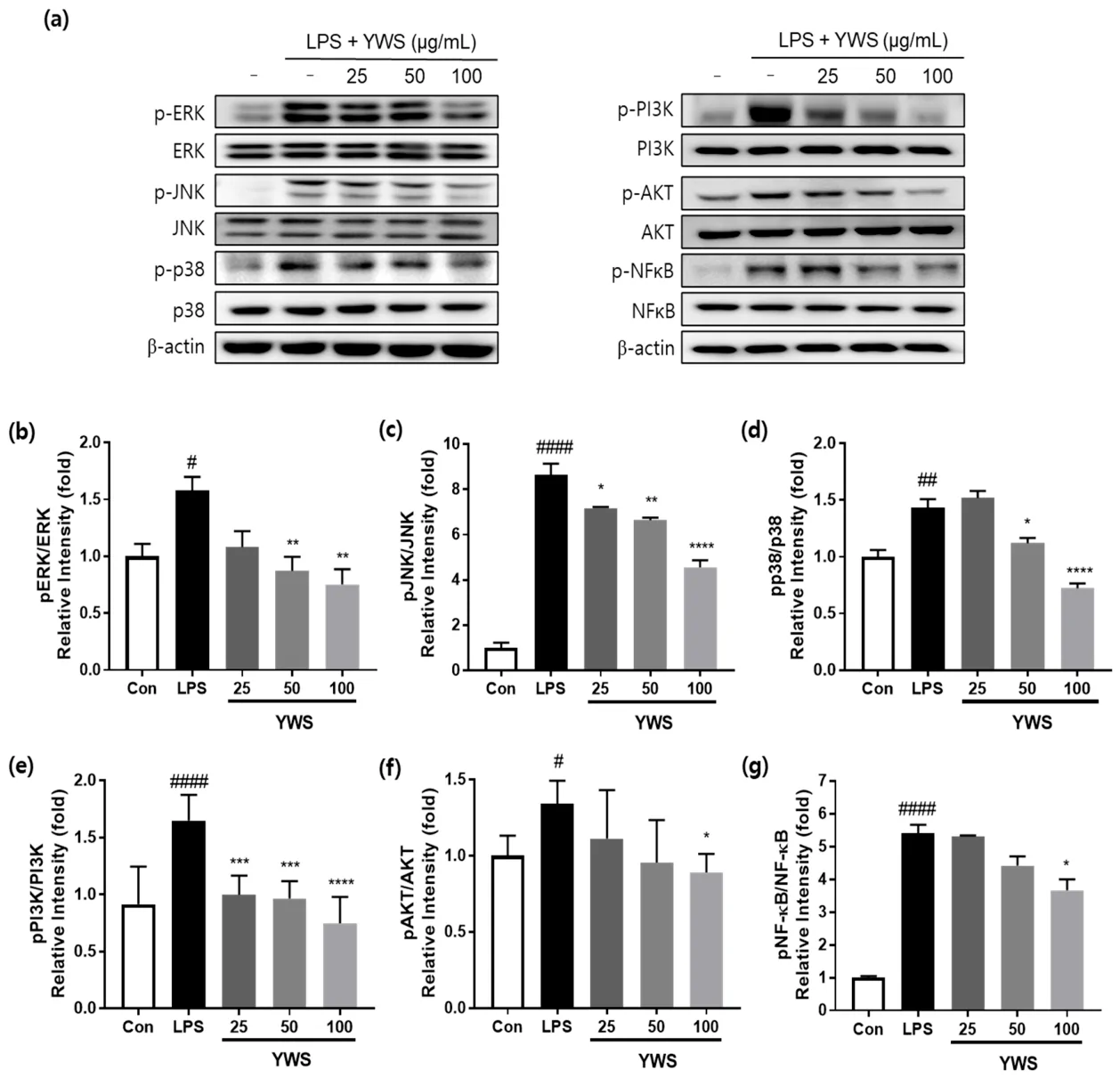
Inhibitory effects of YWS on MAPK and PI3K/Akt pathway activation in LPS-stimulated RAW264.7 macrophages. (**a**) Representative Western blot images showing the expression of total and phosphorylated ERK, JNK, p38, PI3K, Akt, and NF-κB proteins. β-actin was used as a loading control. Quantitative densitometric analyses of phosphorylated (**b**) ERK, (**c**) JNK, (**d**) p38, (**e**) PI3K, (**f**) Akt, and (**g**) NF-κB. Phosphorylation levels were normalized to the corresponding total protein levels and expressed as relative fold changes compared with those in the control group (*n* = 3). # *p* < 0.05, ## *p* < 0.01, #### *p* < 0.0001 vs. untreated control group (Con); * *p* < 0.05, ** *p* < 0.01, *** *p* < 0.001, **** *p* < 0.0001 vs. LPS-treated group (LPS).

To investigate the molecular mechanisms underlying the anti-inflammatory effects of YWS, the activation of MAPK and PI3K/Akt signaling pathways was examined in LPS-stimulated RAW264.7 macrophages. As shown in Figure 3a–d, LPS stimulation markedly increased the phosphorylation of ERK, JNK, and p38, indicating activation of the MAPK pathway; in contrast, YWS treatment significantly attenuated these changes without altering total protein levels. Similarly, LPS stimulation enhanced PI3K and Akt phosphorylation, suggesting activation of the PI3K/Akt pathway (Figure 3e,f). YWS markedly suppressed these effects, but total PI3K and Akt levels remained unchanged. Given that the MAPK and PI3K/Akt pathways contribute to the activation of NF-κB, a key regulator of inflammatory responses, the observed reduction in NF-κB phosphorylation following YWS treatment supports its inhibitory effect on inflammatory signaling. Collectively, these findings indicate that YWS attenuates LPS-induced inflammatory responses in RAW264.7 macrophages by suppressing MAPK- and PI3K/Akt-dependent activation of NF-κB (Figure 3g).

### 2.4. Effect of YWS on Esophageal Mucosal Injury

**Figure 4:**
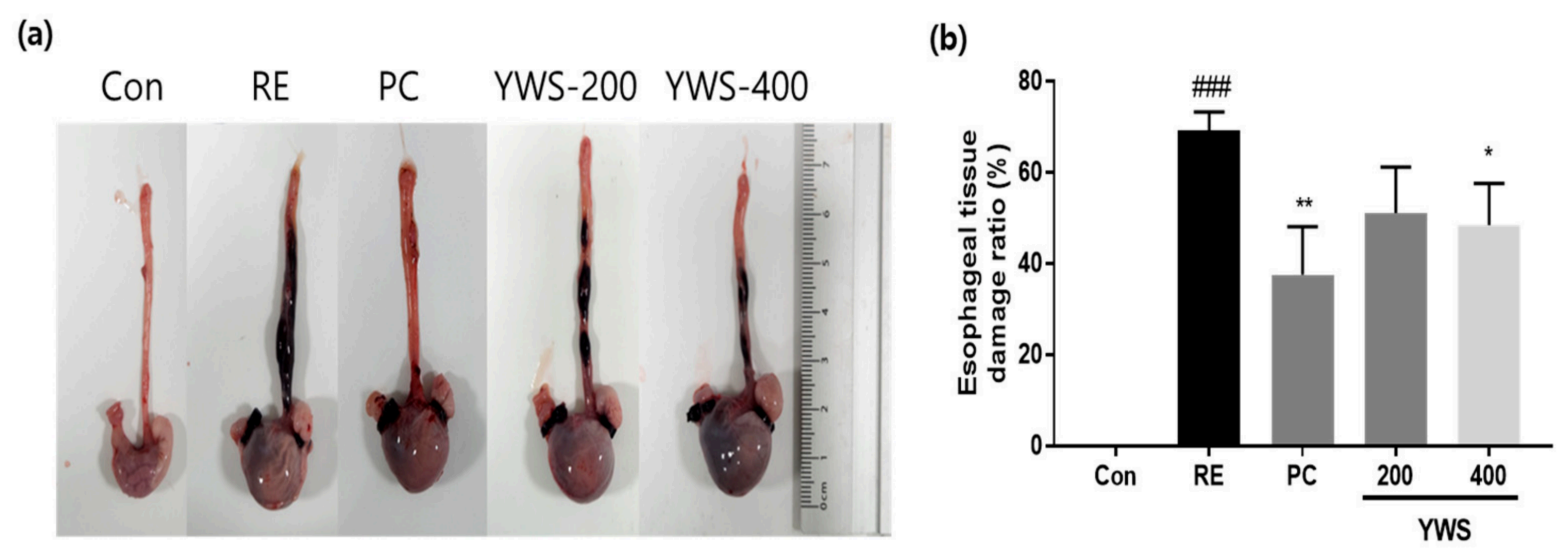
Effects of YWS on gross esophageal injury in rats with reflux esophagitis. (**a**) Representative macroscopic images of esophageal tissues from the sham control (Con), reflux esophagitis (RE), positive control (PC; omeprazole, 20 mg/kg), and YWS-treated groups. (**b**) Quantification of esophageal lesion area expressed as a percentage of the total esophageal mucosal area (*n* = 7). ### *p* < 0.001 vs. sham control group (Con); * *p* < 0.05, ** *p* < 0.01 vs. RE-induced group (RE).

The RE control group exhibited marked gross esophageal mucosal damage and hemorrhage compared with the sham control group (Figure 4a). Treatment with omeprazole (positive control [PC], 20 mg/kg) or YWS reduced the extent of gross esophageal lesions. The lesion area was 69.3% in the RE control group, whereas it was reduced to 37.7% and 51.2% in the PC and YWS 400 mg/kg groups, respectively (Figure 4b). These results demonstrate that YWS attenuated acute reflux-induced esophageal injury.

### 2.5. Histological Effects of YWS

**Figure 5:**
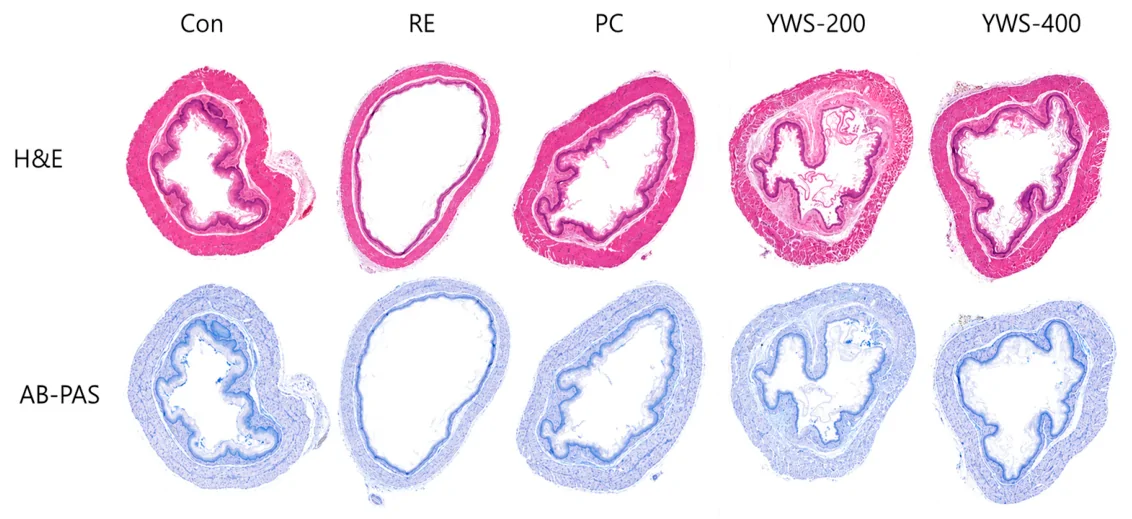
Representative H&E and AB-PAS staining images of esophageal tissues.

Histological examination was performed to assess esophageal tissue integrity and lesion severity. The normal group exhibited a complete, compact, and well-organized esophageal architecture, with intact mucosa, submucosa, and muscularis externa (Figure 5). In contrast, gastric reflux induced severe esophageal damage, characterized by tissue deformation, reduced tissue compactness, and epithelial cell shedding. These histopathological lesions were markedly attenuated following treatment with YWS and PC compared with those in the control group.

### 2.6. Effects of YWS on Oxidative Stress and Inflammatory Responses in Rats with Acute RE

**Figure 6:**
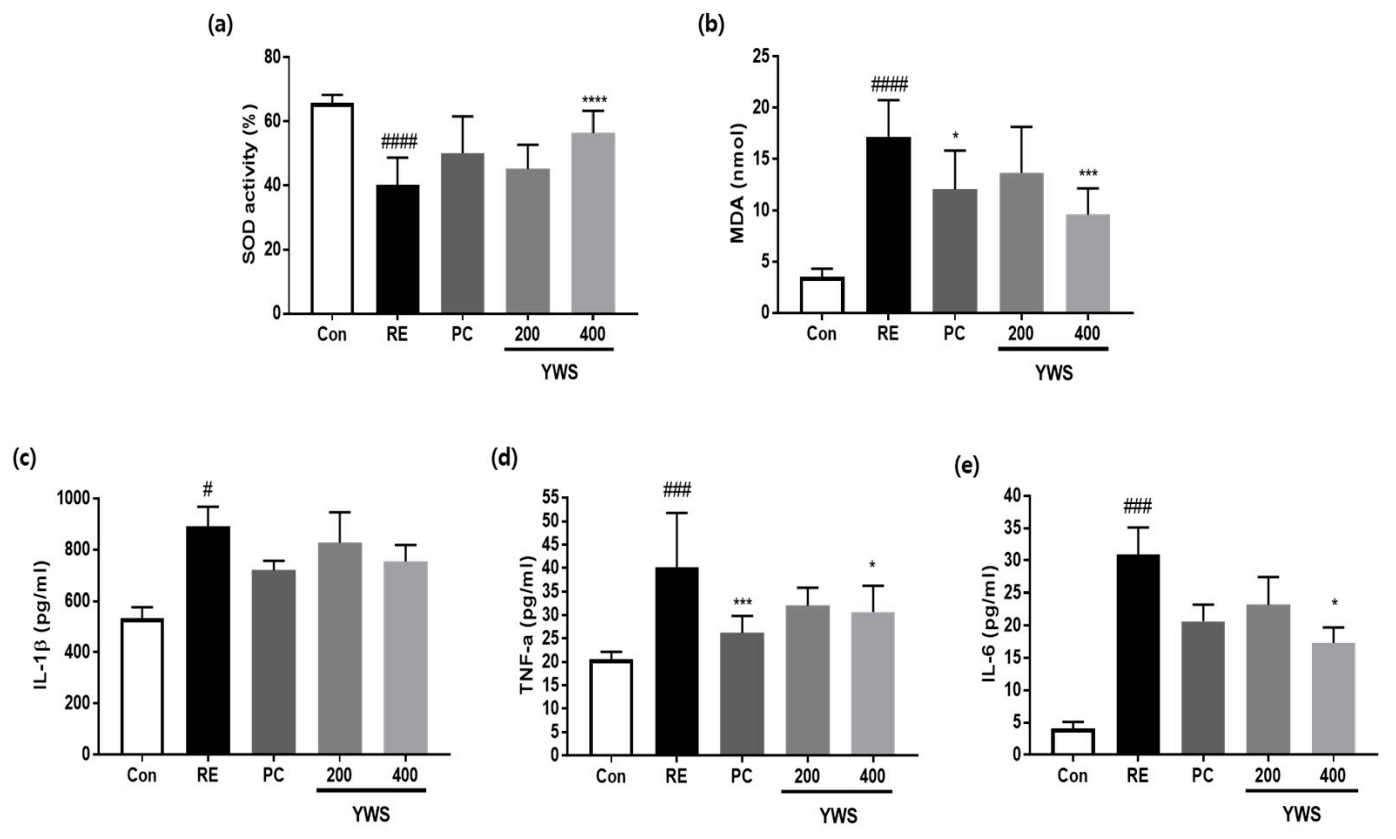
Effects of YWS on oxidative stress and inflammatory parameters. The level of SOD activity (**a**) and MDA levels (**b**) were determined in esophageal tissues (*n* = 7). Serum IL-1β (**c**), tumor necrosis factor (TNF)-α (**d**), and interleukin (IL)-6 (**e**) levels were evaluated in the serum using ELISA. # *p* < 0.05, ### *p* < 0.001, #### *p* < 0.0001 vs. sham control group (Con); * *p* < 0.05, *** *p* < 0.001, **** *p* < 0.0001 vs. RE-induced group (RE).

Serum and esophageal tissue samples were analyzed to evaluate oxidative stress and inflammatory status by assessing malondialdehyde (MDA) levels, superoxide dismutase (SOD) activity, and pro-inflammatory cytokines (IL-6, IL-1β, and TNF-α). As shown in Figure 6a, esophageal SOD activity was markedly decreased in the RE control group relative to the Con group; however, this reduction was significantly reversed following YWS treatment. Conversely, MDA levels in esophageal tissue, as well as IL-6, IL-1β, and TNF-α levels in serum, were substantially elevated in RE control rats (Figure 6b–e). Treatment with YWS significantly reduced these oxidative and inflammatory markers. Collectively, these findings demonstrate that YWS reinforces endogenous antioxidant capacity while suppressing lipid peroxidation induced by oxidative stress. Moreover, the concomitant reduction in pro-inflammatory cytokines indicates that YWS effectively mitigates RE-associated inflammation.

### 2.7. YWS Attenuates Reflux-Associated Activation of IκB/NF-κB Signaling and COX-2 Expression in Esophageal Tissue

**Figure 7:**
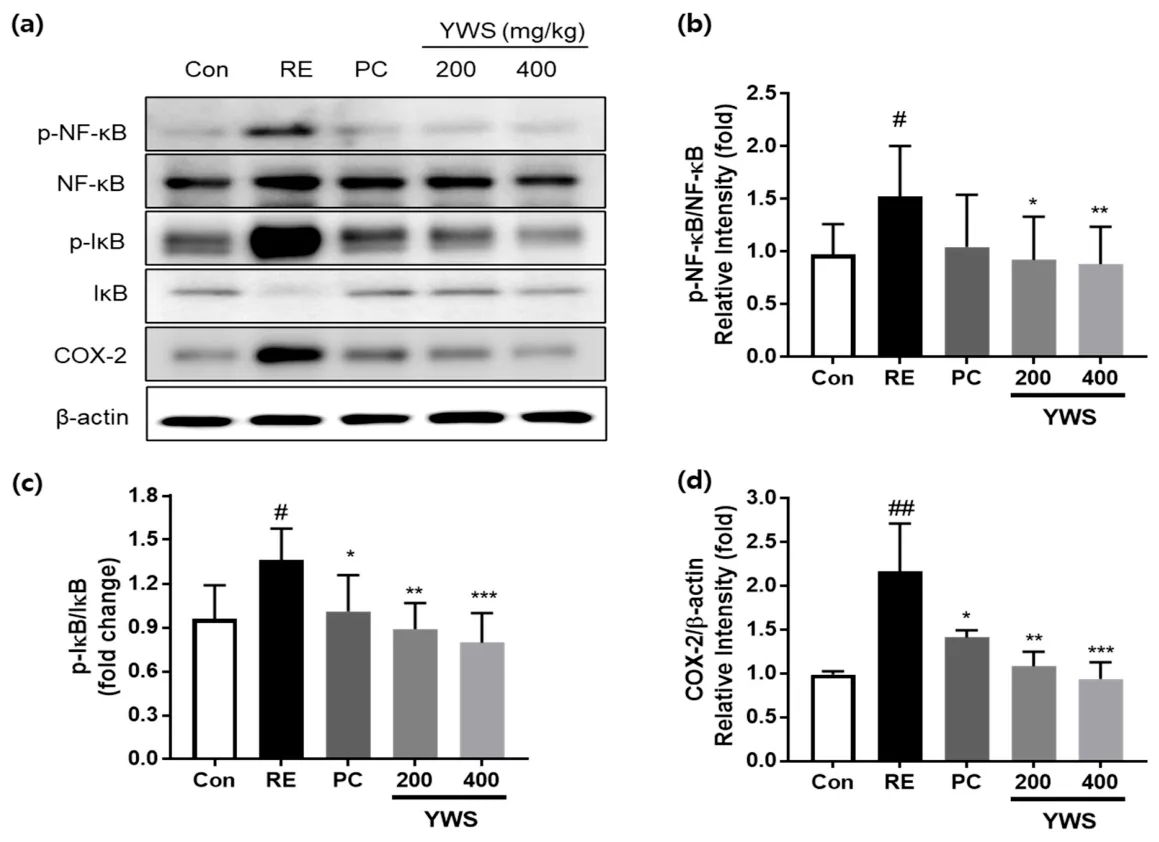
Effects of YWS on NF-κB/IκB signaling and COX-2 expression in whole esophageal tissue homogenates from rats with reflux esophagitis. (**a**) Representative Western blot images of total and phosphorylated NF-κB p65 and IκBα, together with COX-2 protein expression. Quantitative densitometric analyses of (**b**) p-p65/p65, (**c**) p-IκBα/IκBα, and (**d**) COX-2/β-actin. Phosphorylation levels of NF-κB p65 and IκBα were normalized to their corresponding total protein levels, whereas COX-2 expression was normalized to β-actin. Data are expressed as relative fold changes compared with the sham control group. β-actin was used as a loading control. # *p* < 0.05, ## *p* < 0.01 vs. sham control group (Con); * *p* < 0.05, ** *p* < 0.01, *** *p* < 0.001 vs. RE-induced group (RE).

The expression and phosphorylation levels of NF-κB and IκB, along with COX-2 expression, were analyzed in esophageal tissues obtained from RE rats to elucidate the involvement of NF-κB signaling in RE. As shown in Figure 7, phosphorylation of NF-κB and IκB was markedly increased in the RE group compared with the Con group, indicating activation of the NF-κB pathway. COX-2 expression was also significantly increased in the RE group compared with the Con group. In contrast, YWS administration significantly reduced the p-p65/p65 and p-IκBα/IκBα ratios compared with those in the untreated RE group. YWS also decreased COX-2 protein expression in esophageal tissue. Collectively, these findings suggest that the protective effects of YWS are associated with reduced NF-κB/IκB activation and COX-2 expression at the whole-esophageal-tissue level.

## 3. Discussion

In this study, we demonstrated the protective potential of YWS against reflux-induced esophageal injury and elucidated its underlying anti-inflammatory mechanisms through an integrated approach combining network pharmacology and experimental validation.

To this end, network pharmacology analysis was conducted to predict the potential molecular targets and signaling pathways associated with the anti-inflammatory effects of YWS. Network pharmacology analysis identified 23 core hub genes and multiple enriched KEGG pathways, including the PI3K-Akt, MAPK, Ras, HIF-1, and chemokine signaling pathways. Among these, the MAPK and PI3K-Akt pathways were prioritized for subsequent experimental investigation because they were prominently represented in the enrichment analysis and included highly connected hub genes, particularly *MAPK3* and *AKT1*. The MAPK and PI3K-Akt signaling pathways represent critical hubs that integrate upstream extracellular stimuli and transduce intracellular inflammatory signals [13,14]. MAPK signaling, particularly ERK activation, regulates the expression of pro-inflammatory mediators such as TNF-α and COX-2 [13], whereas the PI3K/Akt pathway plays an important role in inflammatory signaling through downstream signaling molecules and transcription factors [14]. Notably, both pathways are functionally associated with NF-κB activation, a central regulator of inflammatory responses, and with inflammatory cytokine production [15,16]. Consistent with this, several identified genes, including *TNF*, *PTGS2*, *CCL2*, *MMP9*, and *BCL2*, were associated with NF-κB-mediated inflammatory responses, while *AKT1* and *MAPK3* represented highly connected components of the PI3K/Akt and MAPK signaling networks, respectively. Furthermore, enriched pathways, including Ras, chemokine, and HIF-1 signaling, act as upstream or parallel modulators that converge on the MAPK and PI3K-Akt axes. These pathways are functionally interconnected with MAPK and PI3K-Akt signaling and collectively contribute to reflux-induced inflammatory amplification and oxidative mucosal injury [17,18,19,20]. Based on these network pharmacology findings and the known functional links among these pathways, MAPK-, PI3K/Akt-, and NF-κB-associated inflammatory signaling were selected for subsequent experimental evaluation [15,21,22]. Although the present experimental validation focused on MAPK, PI3K/Akt, and NF-κB-associated inflammatory signaling, several other highly connected predicted hub genes, including *STAT3*, *HIF1A*, and *EGFR*, were not examined experimentally. These targets may nevertheless contribute to RE pathophysiology through distinct but functionally interconnected mechanisms. STAT3 is a key mediator of cytokine-dependent inflammatory signaling [23], whereas HIF1A is involved in cellular adaptation to hypoxic and oxidative stress [24]. EGFR, in turn, has been implicated in esophageal epithelial proliferation and mucosal repair following reflux-induced injury [25]. Further investigation of STAT3-, HIF-1-, and EGFR-related signaling may provide additional insight into the molecular mechanisms underlying the protective effects of YWS against reflux-induced esophageal injury. Experimental evaluation in LPS-stimulated RAW264.7 macrophages showed that YWS suppressed MAPK and PI3K/Akt activation. In addition, the in vivo analysis focused on NF-κB/IκB signaling and COX-2 expression as a common downstream inflammatory axis in esophageal tissue [16]. Consistent with previous studies demonstrating its antioxidant properties, this study confirmed that YWS effectively inhibited intracellular ROS production in RAW264.7 macrophages. Excessive ROS generation is a key upstream regulator that triggers redox-sensitive inflammatory signaling pathways, including MAPK and PI3K/Akt, and thus a reduction in ROS level can attenuate downstream inflammatory responses [26,27]. These signaling events ultimately lead to the activation of NF-κB, thereby driving the transcriptional upregulation of various inflammatory mediators [28]. Consistent with this mechanism, this study demonstrated that YWS significantly inhibited the production of pro-inflammatory cytokines, including TNF-α and IL-6, in RAW264.7 macrophages. Western blot analysis further revealed that YWS suppressed the activation of MAPKs, PI3K/Akt, and NF-κB, suggesting that its anti-cytokine effects are intrinsically linked to the regulation of these upstream signaling pathways. The anti-inflammatory and protective effects of YWS were further evaluated in vivo using an acute reflux esophagitis model induced by pyloric and forestomach ligation [29]. This model is widely used to assess short-term esophageal mucosal injury, oxidative stress, and inflammatory responses by inducing acute and severe exposure of the esophageal mucosa to refluxed gastric contents. In this model, the accumulation and reflux of gastric contents cause marked esophageal injury, including epithelial erosion, hemorrhage, and inflammatory cell infiltration [29,30]. However, because this model does not fully replicate the chronic and recurrent features of human reflux esophagitis, lower esophageal sphincter dysfunction, or the heterogeneous composition of clinical refluxate, including non-acidic components, the present findings should primarily be interpreted as evidence of protection against acute reflux-induced esophageal injury.

YWS also attenuated reflux-associated oxidative stress and inflammatory responses. Oxidative stress plays a critical role in reflux-induced esophageal injury, characterized by increased ROS and lipid peroxidation, along with decreased activity of antioxidant enzymes such as SOD [30]. In this study, YWS improved oxidative stress status in vivo by decreasing MDA levels and restoring SOD activity. In addition, YWS decreased serum levels of TNF-α, IL-6, and IL-1β and suppressed the activation of the IκB/NF-κB signaling axis and COX-2 expression in esophageal tissues. These in vivo findings complement the cellular results, in which YWS suppressed ROS generation, inflammatory mediator production, and MAPK-, PI3K/Akt-, and NF-κB-related signaling. It should be noted that the in vivo experiments focused on NF-κB/IκB signaling and COX-2 expression as downstream inflammatory responses and did not directly evaluate upstream MAPK or PI3K/Akt phosphorylation in esophageal tissue. Although YWS modulated these pathways in LPS-stimulated macrophages, their contribution to the in vivo protective effects of YWS remains to be established and warrants further evaluation of ERK and Akt phosphorylation in reflux-injured esophageal tissue.

## 4. Materials and Methods

### 4.1. Sample Preparation

YWS was prepared following a previously reported method [12]. YWS extract, six herbal medicines, Glycyrrhizae Radix et Rhizoma, Massa Medicata Fermentata, Phyllostachyos Caulis in Taeniam, Ponciri Fructus Immaturus, Scutellariae Radix, and Ostreae Testa (shell of *Ostrea gigas* Thunberg) were purchased from Kwangmyungdang Medical Herbs (Ulsan, Republic of Korea). *O. gigas* was subjected to water extraction, whereas the other five herbal materials were extracted using 30% ethanol (EtOH). All samples were subjected to reflux extraction, followed by vacuum concentration and subsequent lyophilization to obtain dried extracts. The HPLC fingerprint of YWS is described in our previous report [11].

### 4.2. Integrated Network Pharmacology Analysis

Network pharmacology analysis of YWS was conducted through target prediction, network construction, and functional enrichment analyses. YWS comprises six components: Glycyrrhizae Radix et Rhizoma, Massa Medicata Fermentata, Phyllostachyos Caulis in Taeniam, Ponciri Fructus Immaturus, Scutellariae Radix, and Ostreae Testa (shell of *Ostrea gigas* Thunberg). Because no target genes associated with Ostreae Testa were identified in the databases, Ostreae Testa was excluded from subsequent network pharmacology analyses. Potential human targets of the remaining five herbs were predicted using the PharmMapper (https://www.lilab-ecust.cn/pharmmapper/submitfile.html, accessed on 3 February 2026), SwissTargetPrediction (http://www.swisstargetprediction.ch/, accessed on 5 February 2026), and STITCH (https://stitch-db.org/, accessed on 5 February 2026), and all retrieved targets were standardized using the UniProt database. Simultaneously, targets related to “reflux esophagitis” were collected from GeneCards (https://www.genecards.org/, accessed on 25 February 2026) (relevance score ≥ 5) and OMIM (https://www.omim.org/, accessed on 25 February 2026). After removing duplicates, common targets of YWS and RE were identified using a Venn diagram.

A protein–protein interaction network was constructed using the STRING database to explore the interactions between these targets. The settings were restricted to “*Homo sapiens*” with a minimum required interaction score of 0.400 (medium confidence). The network was visualized and analyzed using Cytoscape (v.3.10.4). Specifically, Degree Centrality and Maximal Clique Centrality (MCC) were calculated via the CytoHubba plugin, and cluster analysis was performed using MCODE. Key hub genes were identified through the intersection of the results of these topological analyses. Finally, Gene Ontology (GO) enrichment analysis—including biological process (BP), cellular component (CC), and molecular function (MF) terms—and Kyoto Encyclopedia of Genes and Genomes (KEGG) pathway enrichment analysis were performed using STRING. Enriched GO terms and KEGG pathways were prioritized based on a false discovery rate (FDR) < 0.01, the inclusion of at least three hub genes, and the exclusion of cancer- and infection-related pathways. The top 20 terms were visualized using R software (v.4.5.2).

### 4.3. Cell Culture

Murine macrophage RAW264.7 cells were sourced from the American Type Culture Collection (ATCC, Manassas, VA, USA). The cells were maintained in Dulbecco’s modified Eagle medium (DMEM; Gibco, Grand Island, NY, USA) supplemented with 10% heat-inactivated fetal bovine serum, 100 U/mL penicillin, and 100 μg/mL streptomycin (Gibco, Grand Island, NY, USA). Cultures were incubated at 37 °C in a humidified incubator with 5% CO_2_. YWS (25–100 μg/mL) and the positive control, NG-monomethyl-L-arginine (NMMA, 50 μM), were added 2 h prior to LPS stimulation. After pretreatment, LPS was added at a final concentration of 0.5 μg/mL, and cells were incubated for another 22 h. Cell viability was assessed using the Cell Counting Kit-8 (CCK-8; Dojindo Molecular Technologies, Kumamoto, Japan) assay. The supernatants were centrifuged at 12,000 rpm for 5 min, and the culture medium was then removed; subsequently, basal medium containing 10% CCK-8 working solution was added to each well. The plates were incubated in the culture chamber for another 2 h. Absorbance was recorded at 450 nm, and viability was calculated as a percentage relative to that of the untreated control (Con).

### 4.4. Measurement of Reactive Oxygen Species (ROS)

RAW264.7 cells were seeded in 96-well plates at a density of 1 × 10^5^ cells per well and incubated for 24 h. YWS was added at concentrations ranging from 25 to 100 μg/mL for 2 h. After pretreatment, LPS was added at a final concentration of 0.5 μg/mL, and cells were incubated for another 22 h. The culture medium was removed, and 1 mL of PBS containing 10 μL DCFH-DA was added to each well; the cells were incubated at 37 °C for 30 min. All culture media were carefully removed, and the cells were washed three times with PBS. Fluorescence intensity was measured using a microplate reader.

### 4.5. Measurement of NO and Pro-Inflammatory Cytokine Production

NO production was determined by quantifying nitrite levels in the culture supernatant using the Griess Reagent System (Promega, Madison, WI, USA). Concentrations of pro-inflammatory cytokines—TNF-α, IL-1β, and IL-6—in culture media or serum were measured with commercially available sandwich ELISA kits (R&D Systems, Minneapolis, MN, USA) following the manufacturers’ protocols.

### 4.6. Animals

Six-week-old male Sprague–Dawley rats were obtained from Orient Bio Inc. (Seongnam, Republic of Korea) and acclimated for seven days prior to the experiment under controlled laboratory conditions (temperature 22 ± 2 °C, 12 h light/dark cycle, humidity 55 ± 10%) with ad libitum access to standard chow and sterilized water. A total of 35 rats were randomly divided into five experimental groups (*n* = 7 per group). The sample size was estimated based on previous studies conducted under similar experimental conditions, in which 7 animals per group were sufficient to detect significant differences between treatments. From three days before the induction of RE until the end of the experiment, YWS was administered orally once daily at doses of 200 or 400 mg/kg/day. Omeprazole (20 mg/kg/day) was administered in the same manner as that in the positive control group. Animals were fasted for 18 h before surgery and had free access to water. Surgical procedures were performed under isoflurane inhalation anesthesia, and the stomach was exposed through a mid-abdominal incision. RE was induced via ligation of the pylorus and forestomach using a nylon tie. Rats in the sham control group (Con) underwent midline laparotomy only, without ligation of the stomach or duodenum [29,30]. After 5 h, followed by routine laparotomy, blood from the abdominal aorta was immediately collected, centrifuged to obtain the supernatant, and stored at −80 °C. The stomach and esophagus were removed to determine the ratio of esophageal injury. The esophageal tissue was stored at −80 °C until analysis. Of note, no animals met the predefined exclusion criteria; therefore, no animals were excluded from the analysis. No animals met the predefined humane endpoint criteria during the study. To minimize bias, investigators responsible for outcome assessments (including biochemical assays, histological evaluation, and data analysis) were blinded to the group allocation throughout the experiment.

### 4.7. Esophageal Mucosal Lesion Ratio and Morphological Analysis of the Esophageal Mucosa

The esophagus was gently washed with saline to remove residual contents and then carefully arranged on white paper. Photographs were obtained and analyzed using ImageJ software (version 1.54g; National Institutes of Health, Bethesda, MD, USA). The gross esophagus tissue damage ratio was calculated as follows: lesion index (%) = [area of esophageal damage (mm^2^)/total area of esophagus (mm^2^)]  ×  100. The calculated value represented the gross mucosal injury ratio. The esophageal tissues were fixed in 4% paraformaldehyde, processed through graded dehydration and clearing steps, and embedded in paraffin. Paraffin blocks were sectioned at a thickness of 5 μm and stained with hematoxylin and eosin (H&E) and Alcian Blue–Periodic Acid Schiff (AB-PAS). Histological features were examined using a Leica light microscope (Leica Microsystems, Wetzlar, Germany).

### 4.8. Western Blot Analysis

Cells and esophageal tissue were lysed using RIPA buffer, and protein concentrations were determined using a BCA protein assay. Equivalent amounts of protein (20 μg per sample) were resolved on 10% SDS–polyacrylamide gels and subsequently transferred onto polyvinylidene difluoride membranes. The membranes were blocked for 1 h at 25 °C with 5% non-fat dry milk dissolved in Tris-buffered saline containing 0.1% Tween-20 (TBST). Membranes were incubated overnight at 4 °C with the appropriate primary antibodies. After several washes with TBST, membranes were exposed to corresponding horseradish peroxidase (HRP)-linked secondary antibodies for 1 h at 25 °C. Protein signals were detected using an enhanced chemiluminescence reagent and visualized on a chemiluminescence imaging system (LAS 4000 mini; GE Healthcare Bio-Sciences, Piscataway, NJ, USA). Densitometric analysis of band intensities was performed using ImageJ software (version 1.54g), and protein expression was normalized to that of β-actin.

### 4.9. Statistical Analysis

Data are expressed as mean ± SD. For in vitro experiments, data were obtained from three independent assays. For multiple comparisons, one-way analysis of variance (ANOVA) with Dunnett’s post-hoc test was used. Statistical significance was set at *p*-value < 0.05 for all experiments. Statistical analyses were performed using a one-way analysis of variance, followed by Dunnett’s post-hoc test to compare each treatment group with the RE group. A *p*-value of <0.05 was considered statistically significant. Analyses were performed using GraphPad Prism version 7.0 (GraphPad Software, La Jolla, CA, USA).

## 5. Conclusions

In summary, our findings suggest that YWS exerts protective effects against acute reflux-induced esophageal injury by attenuating oxidative stress, pro-inflammatory cytokine production, and NF-κB-mediated inflammatory signaling, supporting its potential as a complementary therapeutic candidate for reflux-associated esophageal inflammation. However, because the present study employed a pretreatment regimen in an acute injury model, further studies are needed to determine the therapeutic effects of YWS when administered after disease onset, as well as to evaluate its long-term efficacy using models that more closely recapitulate the chronic and recurrent pathophysiology of reflux esophagitis.

## Data Availability

The original contributions presented in the study are included in the article/Supplementary Material, and further inquiries can be directed to the corresponding author.

## Supplementary Materials

The following supporting information can be downloaded at https://www.mdpi.com/article/10.3390/ph19091422/s1, Table S1: YWS_components_info; Table S2: YSE_Target_gene; Table S3: RE_Target_gene; Table S4: common_target_gene; Table S5: protein–protein interaction; Table S6: Hub_gene_raw_data; Table S7: Hub_gene; Table S8: enrichment_analysis; Figure S1: Enlarged version of the herb-compound–target network shown in Figure 1a.; Figure S2: Enlarged view of the protein–protein interaction (PPI) network of the common targets generated using the STRING database (Figure 1d).
